# An open cohort study of bone metastasis incidence following surgery in breast cancer patients

**DOI:** 10.1186/1471-2407-10-381

**Published:** 2010-07-21

**Authors:** Mitsuru Koizumi, Masataka Yoshimoto, Fujio Kasumi, Takuji Iwase

**Affiliations:** 1 Diagnostic Imaging Group, Molecular Imaging Center, National Institutes of Radiological Sciences, 4-9-1 Anagawa, Inage-ku, Chiba,263-8555 Japan; 2 Department of Nuclear Medicine, Cancer Institute Hospital, 3-8-31, Ariake, Koto-ku, Tokyo 135-8550, Japan; 3 Department of Breast Surgery, Cancer Institute Hospital, 3-8-31, Ariake, Koto-ku, Tokyo 135-8550, Japan; 4 Breast Center, Mita Hospital, International University of Health and Welfare, 1-4-3 Mita, Minato-ku, Tokyo, 108-8329, Japan; 5 Breast Center, Juntendo University, 3-1-3 Hongo, Bunkyo-ku, Tokyo 113-8431, Japan

## Abstract

**Background:**

To help design clinical trials of adjuvant bisphosphonate therapy for breast cancer, the temporal incidence of bone metastasis was investigated in a cohort of patients. We have tried to draw the criteria to use adjuvant bisphosphonate.

**Methods:**

Consecutive breast cancer patients undergoing surgery between 1988 and 1998 (5459 patients) were followed up regarding bone metastasis until December 2006. Patients' characteristics at the time of surgery were analyzed by Cox's method, with bone metastasis as events. Patient groups were assigned according to Cox's analysis, and were judged either to require the adjuvant bisphosphonate or not, using the tentative criteria: high risk (>3% person-year), medium risk (1-3%), and low risk (<1%).

**Results:**

Bone metastasis incidence was constant between 1.0 and 2.8% per person-year more than 10 years. Non-invasive cancer was associated with a very low incidence of bone metastasis (1/436). Multivariate Cox's analysis indicated important factors for bone metastasis were tumor grade (T), nodal grade (pN), and histology. Because T and pN were important factors for bone metastasis prediction, subgroups were made by pTNM stage. Patients at stages IIIA, IIIB and IV had an incidence of >3% per person-year, patients with stage I <1% per person-year, and those with stages II were between 1 and 3%. Further analysis with histology in stage II patients showed that stage IIB with high risk histology also had a high incidence (3% person year), whereas stage IIA with medium risk histology were <1%.

**Conclusions:**

Bone metastasis incidence remained constant for many years. Using pN, T, and histopathology, patients could be classified into high, medium, and low risk groups.

## Background

Bone metastasis is a common complication of breast cancer, with >70% of patients having bone metastasis at autopsy [1]. Despite this high incidence of metastasis found post-mortem, routine bone survey in breast cancer patients is not considered as enough evidence to perform [2]. Two Italian randomized clinical trials failed to prove the usefulness of surveillance that included bone scanning in breast cancer patients [3,4].

Recently, a new treatment involving bisphosphonates for bone metastasis of breast cancer has been developed. Bisphosphonates have evident place in therapy for bone metastasis by reducing bone-related events [5-8]. Bisphosphonates should prevent the development of bone metastasis in breast cancer [9,10]. Gnant et al [9] reported that the administration of bisphosphonate (zoledronic acid) significantly improved disease-free survival. In this study, the use of bisphosphonate was primarily intended to assess the effect of zoledronic acid on bone mineral density. The benefits of bisphosphonate were not confined to the frequency of bone metastasis, but extended to distant and loco-regional metastases in other tissues. Bone metastasis was clearly decreased by the addition of bisphosphonate, even though the difference did not reach significance [9]. Several ongoing large-scale adjuvant bisphosphonate trials have endpoints dealing with disease-free survival and bone metastasis-free survival [10]. Thus, bone-specific therapy in oncology has now been established and is progressing with the advent of bisphosphonates,

For the use of adjuvant with bone-specific drugs, an understanding of temporal incidence of bone metastasis in a specific group of patients is very important, both in planning clinical trials and the actual clinical practice. Therefore, we have investigated the temporal incidence of bone metastasis in subgroups derived from various variables of patient, using data from an open cohort of breast cancer patients that had undergone surgery.

## Methods

### Patients

This prospective study is an open cohort investigation carried out at a single hospital from January 1988 to December 2006, of breast cancer patients that had undergone surgery at the Cancer Institute Hospital in Tokyo, Japan, between January 1988 and December 1998. Patients with any of the following conditions were excluded: (a) bilateral breast cancer in the past or at the time of the surgery, (b) multifocal breast cancer in the ipsilateral breast, (c) bone metastasis diagnosed at the time of surgery or within 30 days after surgery, or (d) patients with incomplete information available on important prognostic factors, such as tumor size and lymph node status. After exclusion on these criteria, 5459 patients were enrolled. The study was approved by the Institutional Review Board and verbal informed consent was obtained from the patients.

### Diagnosis and follow-up

Patients received a physical check every 3 months for first 2 years, every 6 months thereafter up to 10 years after surgery, and subsequently once a year. Bone scans were done to survey for metastases, initially at the time of surgery for staging and once a year thereafter for 5 years. Subsequently, bone scans were done at 7 and 10 years. Scans were also carried out when a physician suspected, or wanted to exclude, the possibility of bone metastasis. When metastasis to other tissues was detected, bone scan was performed to determine the spread of the disease. If the bone scans were positive or equivocal for metastasis, other imaging techniques including X-ray, computed tomography, and magnetic resonance imaging were used to confirm the diagnosis. Recurrence other than in the bone was also determined based on physical examination, chest X-ray, ultrasonography, computed tomography, magnetic resonance imaging and histological examinations. Patients were followed until December 2006.

### Factors analyzed

Several patient characteristics obtained at the time of surgery were used to determine a possible link with bone metastasis; those that could be measured by routine clinical examination are shown in Additional file 1. All factors were converted to categorical variables, these being age at surgery (Age), menstruation state (Mens), breast tumor (T) with minor modification such that T2 tumors were divided into small (2.1-3.0 cm) and large (3.1-5.0 cm), clinical and pathologic lymph node state (N, pN, and axN), histology of breast tumor, status of estrogen (ER) and progesterone receptors (PgR) of the tumor, and adjuvant therapy. Tumors were classified as in situ: non-invasive cancer, T0: no detectable tumor, T1: ≦2.0 cm, T2 small: 2.1-3.0 cm, T2 large: 3.1-5.0 cm, T3: ≧5.1 cm and T4: any size with direct extension to chest wall or skin, according to the 2002 UICC-TNM classification [11]. Histologic classification was performed according to the criteria of The Japanese Breast Cancer Society [12], which differs from the WHO classification. In the Japanese system, invasive ductal carcinoma not otherwise specified (NOS) according to WHO classification system is subdivided into 3 categories based on morphology: papillotubular, solid-tubular and scirrhous carcinomas [12,13]. Lymph node status could be classified by 3 methods according to the 2002 UICC criteria: N: clinical or preoperative classification, pN: pathologic classification and axN: number of axillary nodes involved. Because pathologic classification is more accurate than clinical classification, pN and axN were used for analysis. axN was classified as 1: negative, 2: 1-3 positive nodes, 3: ≧4 positive nodes. pN was taken as a representative lymph node factor, and used for multivariate Cox's analysis. Estrogen receptor status of a tumor was regarded as positive when the concentration was >13 fmol/mg cytosol protein. Progesterone receptor status of tumor was regarded as positive when the concentration was >10 fmol/mg cytosol protein. Adjuvant therapy was classified as no adjuvant, hormone therapy (mainly tamoxifen) only, chemotherapy only, and both hormone therapy and chemotherapy.

pTNM classifications according to the 2002 UICC criteria [11] were also used for data analysis. Since patients with bone metastasis at the time of surgery were omitted from the study, the TpNM classifications used were I, IIA, IIB, IIIA, IIIB, and IV (except for bone metastasis). Because pTNM classification is the result of combined factors, these stages were not used in Cox analysis.

### Analysis procedure and criteria for bone scan recommendation

Data analysis was conducted as follows: first, bone metastasis incidence was calculated in all patients, following which patient characteristics were analyzed to determine those related to the incidence of bone metastasis. Finally, by making subgroups according to characteristics that correlated with increased bone metastasis incidence, the incidence rate in each subgroup was calculated and formed the following tentative criteria for the use of bisphosphonates as adjuvants. The tentative criteria were as follows: patients with a bone metastasis incidence of <1% per person-year group were not recommended for bisphosphonate treatment; patients with a >3% per person-year incidence group were recommended for routine use of bisphosphonates, and patients in the 1 - 3% per person-year incidence group fell into an undetermined grouping in which the use of adjuvant bisphosphonates would be performed at the physician's or patient's discretion.

### Statistical methods

A binominal method was used to calculate the 95% confidence interval for bone metastasis percentage (StatXact version 3.1, SPSS Inc., Chicago, IL). Kaplan-Meier's method was used to compare patient characteristics. The proportionality of each hazard was confirmed by plotting a log-minus-log curve. Cox's proportional hazard regression model was used to calculate the hazard ratio or relative risk by univariate and multivariate analysis. Multivariate Cox's regression analysis was conducted using a forward stepwise method with a likelihood ratio. Factors that were statistically significant in univariate analysis were analyzed by multivariate analysis. In univariate analysis, a p-value of < 0.05 was considered significant. In multivariate analysis, the inclusion criterion was set at p.0.05 and the exclusion criterion was set at p.0.10. Statistical data analysis, except for those, specified ones were conducted with SPSS software, version 11.0 (SPSS Inc.).

## Results

**Figure 1:**
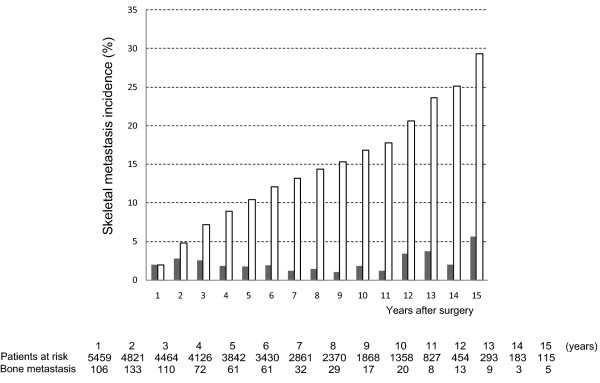
**Annual and Cumulative Incidence of Bone Metastasis for All Breast Cancer Patients**. Annual (shaded bar) and cumulative (open bar) incidence of bone metastasis is given for all patients enrolled, including those with non-invasive breast cancer. The numbers of patients at risk and with bone metastasis events are shown in the lower column.

Additional file 1 shows patient demographics and the percentage of patients who developed bone metastasis. The annual bone metastasis incidence was 1.0-2.8% per person-year for the first 10 years (Figure 1). Very few bone metastases was observed in patients with non-invasive cancer and Paget's disease, and hence data from these patients were omitted from further analysis. Bone metastasis was diagnosed in 690 patients out of the 5023 patients with invasive breast cancer,

Additional file 2 summarizes the results of univariate and multivariate Cox's proportional hazard analysis. The relative risk was calculated for each factor, using a hazard ratio = 1 as a baseline in each classification. In univariate Cox's analysis, age, tumor, nodal state (pN and axillary N), histology, and adjuvant therapy were characteristics that significantly correlated with an increased incidence in bone metastasis. However, menstruation status, ER, and PgR were not significantly correlated. Since both pN and axillary node are lymph node classifications, pN was taken as a representative value of nodal involvement in multivariate analysis. In the multivariate Cox's proportional hazard analysis, age, breast tumor, pN, histology and adjuvant therapy were significantly correlated factors. Multivariate Cox's proportional hazard analysis gave the following results; for pN, the hazard ratios were 2.41 in patients with pN= 1, 5.09 in patients with pN = 2, and 4.92 in patients with pN = 3, taking the value of patients with pN = 0 as 1.0. For tumor size, the hazard ratios were 0.62 in patients with non-palpable tumor, 1.63 in patients with T2 small, 2.04 in patients with T2 large, 2.74 in patients with T3, and 3.25 in patients with T4, taking the value of patients with T1 as 1.0. For histology, the hazard ratios were 2.16 in patients with scirrhous cancer, 2.74 in patients with invasive lobular carcinomas, and 1.49 in patients with solid tubular carcinoma, taking the value of the patients with papillotubular carcinoma as 1.0. Adjuvant therapy was included in multivariate analysis as an adjusting factor. This factor was worse (i.e. giving a high hazard ratio: 1.05 in hormone therapy, 2.88 in chemotherapy, and 2.07 in chemotherapy plus hormone therapy) in univariate analysis, and better (giving a low hazard ratio: 0.64 in hormone therapy, 0.8 in chemotherapy, and 0.66 in chemotherapy plus hormone therapy) in multivariate analysis. This discrepancy could be explained as follows: the high hazard ratio in univariate analysis reflected the use of adjuvant therapy for high risk patients, and the low hazard ratio arose because the patient imbalance was adjusted by other factors, revealing the true effect of adjuvant therapy on bone metastasis development, adjuvant hormone, chemotherapy or both decreasing the risk of bone metastasis.

**Figure 2:**
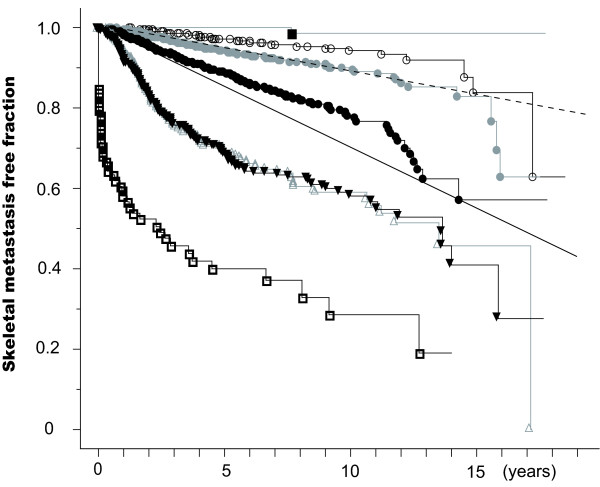
**Bone Metastasis Free Fraction Curves of Breast Cancer Patients According To pTNM Stage**. Bone metastasis free fractions are shown for patients with invasive breast cancer patients according to pTNM stage. Each curve shows the bone metastasis-free fraction of patients as follows: in situ (black square), stage I (open circle), stage IIA (gray circle), stage IIB (black circle), stage IIIA (open triangle), stage IIIB (black inverted triangle), and stage IV other than bone (open square). Point marks indicate events. Oblique straight lines indicate 3% per person-year (solid) and 1% per person-year (dotted) lines.

Figure 2 gives the Kaplan-Meier curves of patients grouped by pTNM stage. Patients with stage I disease had a bone metastasis rate of less than 1% per person-year. Bone metastasis incidence in patients with stage IIA and IIB disease was 1 to 3% per person-year, although stage IIA patients had a significantly lower incidence than stage IIB patients. The incidence of bone metastasis was >3% per person-year in patients with stage IIIA, IIIB, or IV disease.

**Figure 3:**
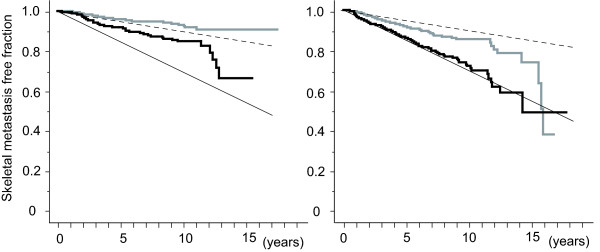
**Bone Metastasis Free Fraction Curves of Stage II Breast Cancer Patients**. Bone metastasis free fractions are shown for stage II breast cancer patients with medium (left) and high risk histology (right). Grey stair-like line indicates stage IIA and black stair-like line indicates stage IIB. Oblique straight lines indicate 3% per person-year (solid) and 1% per person-year (dotted) lines.

Further analysis was conducted on patients with stage II having an incidence of bone metastasis of between 1 and 3% per person-year. Among the significant factors, T, pN, and histology showed a strong correlation with the development of bone metastasis (Additional file 2). pTNM classification includes T and pN information. Histology could be divided into 2 subgroups from the hazard ratio obtained by Cox's analysis (Additional file 2). Judging from the result of Cox's analysis, patients with scirrhous cancer or invasive lobular cancer were assigned to a high risk group, and patients with papillotubular cancer, solid-tubular cancer or another type of cancer were assigned to a medium risk group. Figure 3A shows the incidence of bone metastasis in stage IIA and IIB patients with medium risk histology, and Figure 3B the incidence in stage IIA and IIB patients with high risk histology. The stage IIA patients with low risk histology showed an annual incidence of <1% per person-year, with stage IIB patients having high risk histology showing the annual incidence at or more than 3% per person-year. The remainders were between 1 - 3%.

**Figure 4:**
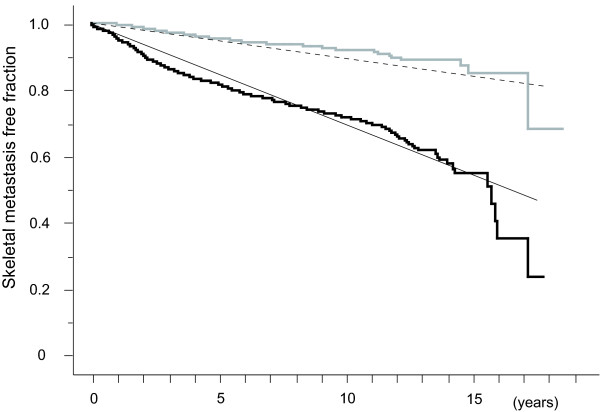
**Bone Metastasis Free Fraction Curves of Invasive Breast Cancer Patients with and without Lymph Nodal Metastasis**. Bone metastasis free fractions are shown for patients with pathological lymph node metastasis (black step-like line) and without (gray step-like line). Oblique straight lines indicate 3% per person-year (solid) and 1% per person-year (dotted) lines.

Since these result seem to be complicated, we sought a simpler classification. Because pN proved to be a very strong factor related to bone metastasis in Cox's analysis, bone metastasis incidence in patients were compared with regard to pN status (positive vs negative). Patients group with positive nodes exhibited an annual bone metastasis incidence of about or >3% per person-year, and patients group with negative nodes an incidence of ~1% (Figure 4).

**Figure 5:**
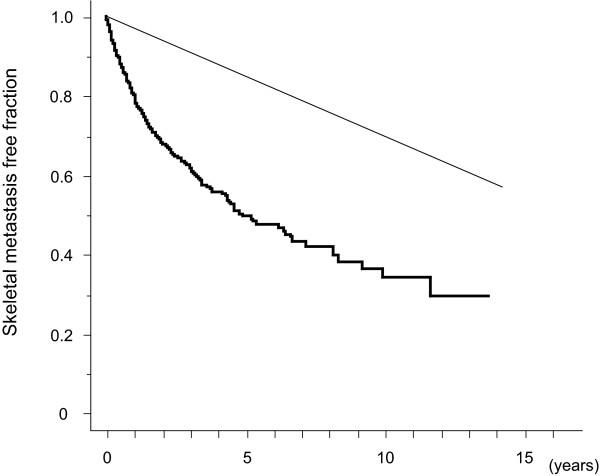
**Bone Metastasis Free Fraction Curve of Patients with Recurrence Other Than Bone**. The bone metastasis free fraction is shown by the black step-like line. Oblique straight lines indicate 3% per person-year (solid) and 1% per person-year (dotted) lines.

The patient group with recurrence of metastasis to tissues other than bone during the follow up period was another high risk group. Figure 5 shows bone metastasis incidence in patients with recurrence of metastasis to a region other than bone. There were 783 patients classified to this group, and 242 patients developed bone metastasis during the follow-up period. This group had an incidence of bone metastasis of >3% per person-year.

Based on the results of this analysis, candidates for adjuvant bisphosphonate therapy are summarized in Additional file 3.

## Discussion

Over 70% of breast cancer patients present bone metastasis at autopsy [1]. Indeed, 63% (555/880) of patients dying of the disease had had bone metastasis detected by scanning. In 1970' and 1980's, bone scan had been considered an essential procedure during both initial staging and regular follow-ups in patients with breast cancer, because of the high incidence of bone metastasis being reported [12-15]. However, a re-evaluation of the procedure in the late 1980's suggested the incidence of bone metastasis was not as great as previously thought [16-19]. The results of Rosseli et al and GIVCO [3,4] indicated that, while periodic survey picked up recurrence more than just a physician's check-up, the overall survival rate did not differ from patients not given a bone scan. Consequently, guidelines were issued indicating that a bone scan was not justified at regular intervals during follow-up [2].

With the development of bisphosphonates, agents that effectively reduce bone- related events caused by bone metastasis, an effective treatment is now available [5-7]. Indeed, recent guidelines for breast cancer treatment strongly recommend bisphosphonate treatment in cases of bone metastasis [8]. These results indicate that early detection of bone metastasis in breast cancer patients can reduce bone-related events and improve quality of life. Regrettably, these studies conducted by Rosseli et al and GIVCO did not investigate bone-related events because this concept was not extant when the two studies were conducted [9,10].

The use of bisphosphonates in preventing the development of bone metastasis is currently under investigation, clodronate and oral bisphosphonate both being investigated as adjuvants. Three large randomized studies have been reported [7,20,21]. Two trials [7,20] gave positive data for the use of clodronate, but one trial [21] was negative. Meta-analysis including these studies revealed no significant difference in overall survival, or in bone metastasis-free survival, in breast cancer patients receiving adjuvant clodronate treatment [22]. Recently the results of more potent bisphosphonate adjuvant, zoledronic acid, were reported by Gnant et al [9], who have reported that its administration was associated with a significant improvement in disease-free survival. In their study, the use of bisphosphonate was primarily intended to assess the effect of zoledronic acid on bone mineral density. The benefits from bisphosphonate were not confined to the frequency of bone metastasis, but extended to all distant and loco-regional metastases. While bone metastasis was indeed decreased by the use of bisphosphonate, this effect was not significantly significant [9]. There are several ongoing large-scale adjuvant bisphosphonate trials looking at the endpoints of disease-free survival and bone metastasis survival [10].

This report presents the temporal incidence of bone metastasis associated with breast cancer using data obtained at a single hospital. Bone metastasis has been diagnosed by several experts using consistent criteria [23,24], and confirmatory studies were carried out by using other imaging methods. Many follow-up bone scan studies in breast cancer have been reported. Unfortunately, most of them had limited numbers of patients, short follow-up times, or both [16-19]. Most studies reported their results for only a 2-year follow-up period after surgery. However, as Figure 1 indicates, the annual incidence of bone metastasis is constant for at least the first 10 years following surgery. Indeed, breast cancer patients who present recurrence after a long disease-free interval can often be observed in daily clinical practice. Our results are consistent with these observations and stress the importance of long-term monitoring.

A number of prognostic factors can be used to estimate survival and disease-free survival of breast cancer patients. These include nodes status, histologic classification of the tumor, nuclear grading of the tumor, tumor size, estrogen and progesterone receptor status, the S-phase fraction, mitotic index, p53, Ki-67, among other parameters [25-28]. Of these prognostic factors, characteristics routinely available to clinical practice were selected for this investigation. Analysis of patient characteristics for correlation with bone metastasis showed that nodal status (pN), tumor size (T) and histology were strongly positively correlated with an increase in the incidence of bone metastasis. Because very few bone metastases developed in patients with non-invasive carcinoma and Paget's disease, surveillance for bone metastasis is not necessary in these patients. Because our study started in 1988, old pathologic classification was used. A serious flaw was therefore the use of Japanese-based pathological classification, which differs from the WHO classification on the point that invasive ductal carcinoma not otherwise specified (NOS) according to WHO classification system was subdivided into 3 categories based on morphology, i.e. papillotubular, solid-tubular and scirrhous carcinomas [12,13]. Pathological criteria could not be used other than that for Japan. However, other factors were able to be translated into the 2002 UICC criteria, so that the present results can be used regardless of the pathological classification of invasive ductal cancer.

In future, the criterion of percentage (person-year) for the use of bisphosphonates to prevent bone metastasis should be required with regard to cost-effectiveness. A report from Breast Cancer Research Group in Ontario, Canada, indicates that tests with a rigid criterion in detecting metastases in <1% of patients have a significant false-positive rate are not clinically useful [29]. Another report indicated that bone survey should be conducted in patient groups whose bone metastasis incidence was 3% [30]. We tentatively adopted 1 to 3% criterion for the adjuvant use of bisphosphonates, which referred to patients in groups with a bone metastasis incidence of <1% per person-year not being recommended for adjuvant bisphosphonate treatment, patients in groups with a >3% per person-year incidence being recommended, and patients in groups with a 1 - 3% per person-year incidence falling into an undetermined area where it is unclear whether the prophylactic use of bisphosphonates can provide any significant benefit. This concept needs to be verified by actual clinical studies. Patients with stage IIA having medium risk histology and stage I showed <1% per person-year incidence are not recommended for adjuvant bisphosphonate therapy. Patients with stage III, stage IV (except for bone), stage IIB with high risk histology, and those with recurrence other than bone showing >3% per person-year incidence, are seen as candidates that should receive adjuvant bisphosphonate therapy. Patients with stage IIB having medium risk histology and IIA having high risk histology had a bone metastasis incidence between 1 and 3% per person-year. From another point of view, patients with positive nodes had an annual bone metastasis incidence >3% per person-year, making these patients the candidates to receive adjuvant bisphosphonate therapy, whereas patients with negative nodes with an annual incidence of ~1% are not recommended the adjuvant treatment. Our data will help improve the design of future clinical studies.

## Conclusions

This study has shown the following. 1) The incidence of bone metastasis is relatively constant for at least 10 years after surgery, with an annual incidence rate of between 1 and 2.8% per person-year. 2) Using tentative criteria based on the annual bone metastasis incidence, patients groups can be classified into those who are and other who are not potential candidates for the adjuvant bisphosphonate therapy. These results should provide an impetus for the adjuvant bisphosphonates as an important intervention in the prevention bone metastasis of breast cancer patients following surgery.

## Competing interests

The authors declare that they have no competing interests.

## Authors' contributions

MK planned the study, undertook data collection, analysis, interpretation, discussion, and writing the manuscript. MY, FK and TI dealt with patients' collection and management, and joined in the planning of the study and its discussion. All authors have read and approved the final manuscript.

## Pre-publication history

The pre-publication history for this paper can be accessed here:

http://www.biomedcentral.com/1471-2407/10/381/prepub

## Supplementary Material

Additional file 1 **Patients' characteristics and incidence of skeletal metastasis**. Additional file 1 shows patient demographics and the percentage of patients who developed bone metastasis. Click here for file

Additional file 2 **Univariate and multivariate Cox's proportional hazard model analysis of factors correlated with incidence of skeletal metastasis**. Additional file 2 summarizes the results of univariate and multivariate Cox's proportional hazard analysis. Click here for file

Additional file 3 **Tentative candidates for adjuvant bisphosphonates**. Additional file 3 illustrates candidates for adjuvant bisphosphonate therapy. Click here for file
